# Utilization of Circulating Tumor Cells in the Management of Solid Tumors

**DOI:** 10.3390/jpm13040694

**Published:** 2023-04-20

**Authors:** Peter C. Kurniali, Michael H. Storandt, Zhaohui Jin

**Affiliations:** 1 Sanford Cancer Center, 701 E Rosser Ave, Bismarck, ND 58501, USA; 2 Department of Internal Medicine, Division of Hematology/Oncology, School of Medicine and Health Sciences, University of North Dakota, Grand Forks, ND 58203, USA; 3 Mayo Clinic, Rochester, MN 55905, USA

**Keywords:** circulating tumor cells, liquid biopsy, ctDNA, solid tumors

## Abstract

Circulating tumor cells (CTCs) are tumor cells shed from the primary tumor into circulation, with clusters of CTCs responsible for cancer metastases. CTC detection and isolation from the bloodstream are based on properties distinguishing CTCs from normal blood cells. Current CTC detection techniques can be divided into two main categories: label dependent, which depends upon antibodies that selectively bind cell surface antigens present on CTCs, or label-independent detection, which is detection based on the size, deformability, and biophysical properties of CTCs. CTCs may play significant roles in cancer screening, diagnosis, treatment navigation, including prognostication and precision medicine, and surveillance. In cancer screening, capturing and evaluating CTCs from peripheral blood could be a strategy to detect cancer at its earliest stage. Cancer diagnosis using liquid biopsy could also have tremendous benefits. Full utilization of CTCs in the clinical management of malignancies may be feasible in the near future; however, several challenges still exist. CTC assays currently lack adequate sensitivity, especially in early-stage solid malignancies, due to low numbers of detectable CTCs. As assays improve and more trials evaluate the clinical utility of CTC detection in guiding therapies, we anticipate increased use in cancer management.

## 1. Introduction

Cancer has emerged as the leading cause of death in the United States and the world. It is projected that 1,958,310 new cancer cases will be diagnosed—and more than 600,000 Americans will die from cancer—in 2023 [1,2,3]. However, in contrast with other leading causes of death, cancer-related mortality continues to decline. From 1991 to 2020, there was an overall reduction in cancer death by 33% [3]. Many factors, including effective cancer screening, better diagnostic tools, the advancement of surgical and radiation techniques, as well as emerging systemic treatments, including chemotherapy, immunotherapy, and targeted therapies, are believed to be the reason behind the decrease in cancer-related mortality. The ability to detect cancer as early as possible, personalize cancer treatments, and effective strategies to prevent or reduce the risk of metastasis will be key in further reducing cancer-related mortality.

Most solid tumor diagnoses are established based on radiographic findings, physical examination, or direct visualization and confirmed by pathologic findings with tissue biopsy. An alternative method to detect cancer is a liquid biopsy. Circulating tumor cells (CTCs) and circulating tumor DNA (ctDNA) are the two main biomarkers detected in liquid biopsies. Although CTCs and ctDNA were identified in 1869 and 1948, respectively, they were not utilized until recently [4].

CTCs are tumor cells that are shed from the primary tumor into circulation, and clusters are responsible for cancer metastases [5]. CTCs evaluation may play an integral role in cancer management in the future. In this review, we discuss CTC detection assays and current evidence regarding the implementation of the detection of CTCs into clinical practice.

This article reviewed the potential utilization of CTCs in solid tumors, including cancer screening and diagnostic, treatment guidance, and cancer surveillance. We reviewed and compiled the pertinent past and ongoing studies on CTCs and solid tumors that will have impacts on the future direction of CTC application.

## 2. How Are CTCs Being Detected and Isolated?

Detection and isolation of CTCs from the bloodstream are based on properties that distinguish CTCs from normal blood cells. Current CTC detection techniques can be divided into two main categories: label-dependent or label-independent detection (Table 1) [6].

Label-dependent isolation methods depend upon antibodies that selectively bind cell surface antigens present in CTCs. Unlike blood cells, CTCs express epithelial markers, including epithelial cell adhesion molecules (EpCAM) and cytokeratin (CK) [7,8]. Specific techniques utilizing this principle include antibody conjugation to magnetic nanoparticles and microfluidics [9,10]. Although the phenotypic and functional definitions of CTCs vary between multiple studies, it is widely accepted that CTCs lack CD45 and express EpCAM and CK [11]. The cytokeratin expression pattern may differ in various cancer types and will typically mimic the expression pattern in tissue biopsy specimens; moreover, it may change as patients receive treatment or have progression. CellSearch is one main technology utilizing antibody conjugation to magnetic nanoparticles to isolate CTCs, and among the multiple kits produced, one has received Food and Drug Administration (FDA) approval. This technology depends upon the binding of anti-EpCAM-ferrofluid to epithelial CTCs with subsequent isolation of labeled cells via magnets [9]. Although EpCAM is universally expressed by CTCs, the expression may change after cancer progression or during cancer treatment. Therefore, this assay is limited by low sensitivity and the downregulation of epithelial markers on the cell surface as tumor cells become malignant and undergo epithelial to mesenchymal transformation [9,12]. Microfluidics depends upon controlled fluid flow which allows for optimization of cell contact with the walls of a microchip, which have a relatively high surface area and are coated with antibodies for CTC isolation, allowing for the binding of cell surface antigens to the chip walls, while other cells continue to move through the microchip [10]. This assay allows for high sensitivity while maintaining high cell viability; however, it is limited by its ability to process only small sample volumes. 

Label-independent detection can isolate CTCs not based on cell surface markers but rather on the size, deformability, and biophysical properties of CTCs [6,13]. Methods for isolation include filtration, microfluidics (not dependent on antibodies), density gradient separation, imaging, dielectrophoresis, and inertial focusing [6]. Filtration is dependent upon the fact that CTCs are larger than blood cells, and this technique utilizes membranes with varying pore sizes for the isolation of CTCs [14]. Microfluidics can also be used in the case of size-based separation as well, depending upon the geometry of the CTCs, to allow for the isolation of CTCs from blood cells [10]. Density gradient separation utilizes centrifugation to isolate CTCs from other blood cells, although this assay may be limited by CTCs loss as aggregates form altering density [6,15]. Fiber-optic array laser-scanning technology is a visualization technique that uses high-speed scanning, allowing for the localization of CTCs [16]. Dielectrophoresis uses a non-uniform electric field to polarize cells, allowing tumor cell isolation [13]. Inertial focusing utilizes a complex process involving fluid inertia in microchannels to allow for the focusing of cell populations and collection [6]. Overall, these techniques represent alternative methods to CTCs isolation that do not depend on the recognition of epithelial cell surface markers but rather on differing properties of CTCs to separate them from blood cells.

## 3. Cancer Screening

Current cancer screening methods utilize radiographic technology (mammogram and CT), direct visualization (colonoscopy), stool DNA (Cologuard), and cancer cell analysis (pap smear). However, capturing and evaluating CTCs from peripheral blood could be a strategy to detect cancer at its earliest stage. As demonstrated by Barriere et al. in their analysis of de-differentiated CTCs in early-stage breast cancer, CTCs were detected in 39% of the cohort, suggesting that CTCs are detected early in oncogenesis, prior to the development of metastatic disease, which suggests a role for screening for early-stage malignancies [17].

At this time, much of our understanding of CTC detection for cancer screening has been studied in the context of lung cancer [18]. An early study published in 2014 evaluated CTC detection in conjunction with low-dose computed tomography (LDCT) for patients with chronic obstructive pulmonary disease (COPD) and found that in 168 patients, CTCs were detected in 3%. Of these patients, LDCT detected lung nodules within four years, which were surgically resected, and all were found to be lung cancer [19]. These initial results were promising; however, a subsequent prospective cohort study including 614 adults with COPD eligible for lung cancer screening analyzed CTCs via the size method and found that at baseline screening, CTCs detection was only 26.3% sensitive for the detection of lung cancer [20]. Therefore, while current methodologies have not proved to be clinically effective in lung cancer screening, hope remains that as technological advances improve the sensitivity of these assays, CTC detection may play a role in lung cancer screening [21].

Multiple additional studies have evaluated the role of CTCs assays in cancer screening. In the ICELLATE2 study, 3388 participants without known cancer who had risk factors for cancer development underwent screening for the presence of CTCs, among which 3% had at least 1 CTCs identified in their blood sample, which the authors note is comparable to rates of cancer in the general population [22]. However, rates of malignancy in each group were not reported. In an observational study including 542 patients, of which 277 had a known cancer diagnosis, including a wide range of solid and hematologic malignancies, as well as 265 with risk factors for malignancy but no known cancer diagnosis, CTCs were detected via isolation by size in 100% of patients with cancer and 50% of patients without known malignancy [23]. Of those without known malignancy who had CTCs detected, 20% were found to have an early-stage malignancy via follow-up studies within the following six months.

Additional studies have combined CTC assays in conjunction with conventional cancer screening mechanisms. One study evaluating CTC detection in combination with a prostate-specific antigen (PSA) for the detection of prostate cancer included 20 men with a known diagnosis of prostate cancer, of which 100% were CTCs and PSA positive. Among 27 men undergoing screening for prostate cancer, 20 had both CTCs and PSA positivity, with prostate cancer detected via prostate specific-membrane antigen positron emission tomography (PSMA-PET) in 20/20 [24].

In the near future, it is plausible that the detection of CTCs in peripheral blood may be incorporated into cancer screening. However, at this time, study results are mixed but demonstrate a need for improved sensitivity prior to the mainstream application of CTCs detection for cancer screening. Additionally, more robust studies will be needed to determine a meaningful level of CTCs in the blood, as not all detected CTCs will develop into clinically meaningful cancer.

## 4. Cancer Diagnosis

The current standard to diagnose a solid tumor is through tissue biopsy. However, tissue biopsy techniques face different challenges due to their invasiveness, cost, time, and sampling challenges due to tissue heterogeneity, sample contamination, and the presence of necrotic, fibrotic, and normal tissue [25]. Liquid biopsy is achieved via the sampling of blood or other body fluid to detect malignant cells (CTCs) and tumor DNA (ctDNA). Since blood is directly in contact with most solid tumors, liquid biopsy primarily involves blood sampling [26].

The use of CTCs detection for cancer diagnosis has been most extensively studied in lung cancer. In a study evaluating CTC isolation by size method in 60 patients with lung malignancy and 17 with benign lung lesions, malignant circulating cells were detected in 90% of patients with malignancy and 5% without malignancy [27]. In 72% of patients with lung malignancy who had CTCs detected, the cells allowed for a specific histologic diagnosis. An additional study evaluating the diagnostic utility of CTCs for lung cancer enrolled 75 patients with pulmonary nodules concerning for malignancy, with CTCs detected in a 3 mL sample of blood in 47/67 diagnosed with malignancy (including 69% of primary lung cancers and 75% of lung metastases from extrapulmonary malignancy), and in 0/8 patients with benign lesions [28]. Overall, this study reported a sensitivity of 70% and specificity of 100%, although this was a relatively small sample size.

The diagnostic application of CTCs has been evaluated in other malignancies. In the case of breast cancer, a study enrolled 130 patients with breast cancer, 236 with benign breast disease confirmed by biopsy, and 29 healthy volunteer controls—with CTCs detected in 85% with breast cancer, 16% with benign breast disease, and 3% of controls [29]. When a CTC cutoff value of 2CTCs/4 mL blood was set, sensitivity was 75.56%, and specificity was 95.4%. In the case of prostate cancer, a study including 155 treatment-naive patients with prostate cancer and 98 patients who had not undergone biopsy for suspicion of prostate cancer, utilizing PSA, CTCs positivity, and a 12-gene panel via RNA extracted from CTCs, they were able to predict clinically significant prostate cancer with an area under the curve (AUC) of 0.927 [30].

Overall, liquid biopsy does have its advantages, as it is minimally invasive and has the possibility to detect tumors at an earlier stage. However, disadvantages remain, including lack of sensitivity and inability to perform more specialized histologic analysis, such as determining tumor grade, lymphatic and vascular invasion, etc. However, as these traditional histologic features are replaced by genomic and next-generation sequencing analysis in the future, the utility of liquid biopsy will continue to increase.

## 5. Treatment Navigation (Prognostication and Treatment Guidance) and Precision Medicine

Tumor markers, radiographic, and clinical findings have been traditionally used to predict treatment response. For example, in patients with pancreatic cancer, normalization of previously elevated tumor marker CA-19-9 has been associated with a positive treatment response [31]. The lack of sensitivity and specificity of tumor markers has pushed the development of alternative non-invasive methods to monitor treatment response and to navigate cancer treatment, including CTCs.

In addition to monitoring for treatment response, precision medicine, using tumor markers to guide therapeutic options, has come to the forefront of cancer therapeutics. Next-generation sequencing (NGS) has historically been accomplished via tissue obtained from a tumor biopsy. Peripheral blood samples for NGS have been used in conjunction with or as an alternative to tissue specimens. As CTC isolation is being perfected, a larger number of CTCs will be isolated and may provide a better sample for NGS analysis.

Additionally, during disease progression, cancer cells are expected to acquire new mutations that lead to resistance to cancer treatment. Ideally, we want to routinely obtain tissue samples and repeat NGS with every progression. However, it would be logistically challenging to perform a biopsy at every progression. Therefore, the ability to have a reliable liquid biopsy will be a tremendous benefit.

CTCs analysis has been evaluated in prognostication and treatment guidance of multiple solid malignancies, including breast, prostate, renal cell, non-small cell lung, small cell lung, hepatocellular, pancreatic, gastric, and colorectal [32].

Table 2 summarizes pertinent studies that evaluated the clinical application of CTCs, including the role of CTCs in prognostication and treatment guidance of select solid malignancies.

## 6. The Clinical Application of CTCs in Solid Tumors

### 6.1. Breast Cancer

The clinical utility of CTC detection and its use in prognostication, tailoring therapies based on CTC characteristics, and guiding treatment based on CTC response to therapy has been most extensively evaluated in breast cancer at this time.

#### 6.1.1. Prognostication

Many studies have been conducted evaluating the prognostic value of detectable CTCs at breast cancer diagnosis, both in localized disease and metastatic disease, demonstrating worse progression-free survival (PFS) and overall survival (OS) when compared to patients with undetectable levels or below a threshold of 5 CTCs/7.5 mL whole blood [39,40,41,42,43,44]. This negative prognostic value holds true after initiation of treatment, with worse PFS and OS when levels remain elevated despite therapy [40].

Beyond simply quantifying CTCs, molecular analysis of these cells can provide additional prognostic information. As previously mentioned, as malignant epithelial cells become metastatic, they will undergo epithelial to mesenchymal transition in which they undergo a phenotypic transformation allowing for tissue invasion. In a study of 427 patients with breast cancer, polymerase chain reaction (PCR) was able to detect epithelial to mesenchymal transcription factors within CTCs of 18% of patients, with these patients manifesting a shorter disease-free survival [45]. A subsequent study evaluating the presence of TWIST1, a marker of epithelial to mesenchymal transition, in patients with EpCAM-positive CTCs, found a correlation with shorter overall survival [46]. Beyond transcription factors, others have evaluated surface marker expression on CTCs, with one study finding an association of high expression of CD47 and/or PD-L1 with shorter PFS [47].

#### 6.1.2. Precision Medicine

CTCs can additionally play a role in guiding targeted therapies. With breast cancer, CTCs are able to be evaluated for HER2 expression, which can be dynamic over time [48]. In a study evaluating HER2 expression in CTCs of patients with advanced-stage breast cancer, those with ≥2 HER2 + CTCs/8 mL who received anti-HER2 therapy had longer PFS compared to those not receiving HER2-directed therapy [49].

However, the discrepancy between the HER2 status of the primary tumor and CTCs has been observed [44,50]. As such, multiple studies have sought to determine the therapeutic implications of this discrepancy. In a multicenter phase 2 trial that included seven patients with HER2 non-amplified tumors who had ≥2CTCs/7.5 mL having at least 50% HER2 positivity, treatment with lapatinib, a HER2-directed therapy, resulted in objective response in 0 patients [51]. A similar study conducted using trastuzumab-emtansine in a similar population of 11 patients with metastatic HER2-negative breast cancer with HER2 amplified CTCs (at least 1 HER2 amplified CTCs/7.5 mL) showed partial response in only one patient [52]. The lack of efficacy of HER2-directed therapies in patients with HER2-expressing CTCs with negative expression in the primary tumor was shown in an additional phase II trial including 20 patients, with only partial response observed in 1 patient [33]. Why there is a discrepancy in HER2 status between the primary tumor and CTCs remains unclear. Prior study has shown discordance between the HER2 status of a primary tumor and metastatic disease infrequently [53]. Different hypotheses have been proposed regarding this discrepancy, including the acquisition of HER2 amplification during the mesenchymal to epithelial transformation or heterogeneity of HER2 status within the primary tumor reflected in heterogeneous CTCs [51]. At this time, evidence is lacking in support of tailoring therapy based on CTCs when discrepant from the primary malignancy, although this does remain an area of interest and will require additional study.

An additional application of CTCs in guiding targeted therapy includes single-cell CTC genomic DNA sequencing to detect mutations in the *ESR1* gene, as mutations have been associated with resistance to estrogen deprivation therapy [54,55]. In a study of 46 patients with luminal breast cancer, *ESR1* mutations were detected via sequencing of genomic DNA in the CTCs of 12 patients, all of whom had been treated with estrogen deprivation therapy [54]. However, these mutations were absent in the primary tumor tissue sample but were detected in metastases obtained after CTCs analysis. A study evaluating the detection of *ESR1* mutations in CTCs found concordance with ctDNA in 95% of cases [56]. However, an additional study suggested that the detection of *ESR1* mutations and splice variants may be less sensitive in CTCs compared to ctDNA, although this was a smaller study [57]. In another study evaluating *ESR1* methylation, high concordance was found between detection in CTCs and ctDNA, with methylation associated with poor response to everolimus/exemestane [58].

Within breast cancer, other markers can be evaluated to help guide therapy. For example, multiple studies have demonstrated the ability to detect *PIK3CA* mutations within CTCs, and as mutations in PIK3CA can predict resistance to HER2-directed therapies, this is a potential mechanism to guide treatment [59]. Much like HER2 expression, there may be a discrepancy in mutational status between the primary tumor and CTCs [60]. At this time, to our knowledge, tailoring treatment based on the assessment of *PIK3CA* mutational status in CTCs has not been clinically evaluated.

#### 6.1.3. Treatment Guidance

Multiple studies have demonstrated the negative prognostic value of persistently elevated CTCs in spite of systemic chemotherapy [61,62,63]. Based on this observation, multiple clinical trials have been conducted with the intention of changing therapeutic strategy based on CTCs elevation, essentially allowing CTCs response to guide therapeutic decisions.

SWOG S0500 was an early trial that evaluated 288 women with metastatic breast cancer with CTCs elevation at baseline. Of these, 123 had persistently elevated CTCs after 21 days of chemotherapy and were randomly assigned to continue the current chemotherapy or change to a different chemotherapy [34]. Ultimately, there was no difference in OS observed between these groups. EORTC 90091-10093 BIG 1-12 Treat CTCs was a similarly designed phase II trial, enrolling 63 patients with non-HER2 amplified breast cancer who had at least 1 CTCs/15 mL following neoadjuvant chemotherapy and surgery, and randomized them to receive trastuzumab-based on the prior observation that patients with non-HER2 amplified breast cancer received a benefit from trastuzumab [64]—with 1 hypothesis that this benefit may stem from the targeting of HER2 positive CTCs, versus observation, with a primary endpoint of the rate of detection of CTCs at 18 weeks [65]. However, this study was discontinued early due to futility.

Later, CirCe01 was a prospective, multicenter randomized trial in which 204 patients with metastatic breast cancer who had progression on two prior lines of therapy were enrolled [66]. Patients with elevated CTCs were randomized to standard care or a CTCs-driven arm in which the change in CTCs suggestive of treatment failure would prompt a transition to the next line of therapy. OS was ultimately not different between the two arms, although this study was limited by patient accrual and compliance.

STIC CTCs was a randomized, open-label phase 3 noninferiority trial enrolling women with hormone receptor-positive, ERBB2-negative metastatic breast cancer who were randomized to either clinician-driven first-line treatment versus treatment based on CTC count, with those with CTCs ≥ 5/7.5 mL receiving chemotherapy and CTCs < 5 receiving endocrine therapy [35]. A primary endpoint of noninferiority was achieved. However, a higher rate of chemotherapy-related adverse events was seen in the CTC-driven arm.

Overall, CTC monitoring for treatment guidance remains an area of interest in breast cancer. However, at this time, clinical data are lacking in support of its routine use. Additional clinical trials will be necessary going forward to determine its value in breast cancer management.

### 6.2. Prostate Cancer

Within the field of prostate cancer, CTCs have shown value in the prognostication of patient outcomes. Beyond this, there has been great interest in monitoring androgen receptor splice variants (AR-V) within CTCs to assist in the guidance of therapy.

#### 6.2.1. Prognostication

Much like breast cancer, the elevation of CTCs has been demonstrated to have a negative prognostic value in prostate cancer. Studies have demonstrated poorer PFS and OS among patients with metastatic castration-resistant prostate cancer (MCRPC) [67,68], as well as patients with castration-sensitive prostate cancer [69,70]. Additionally, beyond the initial elevation of CTCs before therapy, multiple studies had shown the persistence of negative prognostic value when the CTCs were continually elevated despite chemotherapy [71,72,73].

Beyond simple detection and quantification, CTCs have been evaluated for various markers, allowing for better prediction of patient outcomes. AR-Vs are one such marker. For example, AR-V7 detected in CTCs has been associated with more aggressive and advanced disease and poorer patient outcomes [74,75]. Another study aimed at determining other prognostic markers in prostate cancer evaluated transcriptional profiles of patients with MCRPC and was able to identify two distinct transcriptional clusters, one of which was associated with worse OS [76]. Additionally, CTC detection has been evaluated in combination with other makers. For example, a study of 711 patients with MCRPC found the combination of CTCs ≥ 5CTCs/7.5 mL with LDH > 250 U/L after 12 weeks of therapy was predictive of 2-year survival of 2% versus 46% observed in patients lacking these markers [77].

#### 6.2.2. Precision Medicine

Much interest has been generated in the evaluation of AR-Vs present in CTCs and the impact of these splice variants on patient response to therapy. A study evaluating the expression of AR-Vs in CTCs from 118 patients with metastatic prostate cancer undergoing treatment with cabazitaxel found that CTCs reduction to <5CTCs was less frequently observed in patients with AR-V9 positive CTCs at baseline and that those with AR-V1 expression after two weeks of therapy exhibited worse OS [78]. An additional study evaluating outcomes of patients with MCRPC who were on their second line or greater of therapy found that those with CTCs with detectable AR-V7 trended towards superior survival when treated with taxanes over an androgen receptor signaling inhibitor (ARSI), whereas those not expressing AR-V7 had superior survival with an ARSI over taxanes [79]. However, another phase II study (PROPHECY) conducted in 118 men with metastatic prostate cancer found that AR-V7 positivity in CTCs prior to treatment with abiraterone or enzalutamide was associated with worse PFS and OS [36]. These studies demonstrate the potential utilization of CTCs for AR-V analysis, which may assist with the selection of therapy.

Other studies have evaluated different markers and their predictive value for response to therapy. A recent phase IB/II study evaluating ribociclib plus docetaxel in MCRPC found non-amplified MYC in baseline CTCs to be associated with longer radiographic PFS [80]. Another phase 2 trial evaluated BIND-014, a prostate-specific membrane antigen (PSMA)-directed docetaxel-containing nanoparticle, and found that after treatment, there was a selective reduction in PSMA-positive CTCs [81], which suggests a role for treatment monitoring of various marker expression in CTCs in guiding further therapeutic choices.

### 6.3. Non-Small Cell and Small Cell Lung Cancer

#### 6.3.1. Prognostication

Within non-small cell lung cancer (NSCLC), multiple studies have demonstrated the negative prognostic implications of elevated CTCs in patients with early-stage disease [82,83,84], with elevation following surgical resection also predictive of poorer prognosis and earlier disease recurrence [85,86,87]. The negative prognostic value also holds true in patients with advanced disease [88,89], including those with persistently positive in spite of treatment [90]. In addition to simple quantification, a recent study utilizing PCR was able to evaluate gene expression and identify a genetic panel predictive of poorer prognosis in NSCLC [91].

Beyond CTCs sampling from peripheral blood, 1 study evaluated CTC detection within the pulmonary venous system and found that in 100 patients with early-stage NSCLC, pulmonary venous CTCs were detected in 48% and associated with poorer PFS [92]. Interestingly, a recent trial randomized patients with early-stage lung cancer undergoing lobectomy to a vein-first (ligation of effluent vessels completed first) or artery first-procedure and found that those who had artery-first ligation had a higher risk of CTC increase during surgery, with a propensity-matched analysis demonstrating better 5-year overall survival in the vein-first group [93]. This demonstrates a role for the understanding of CTC physiology contributing to the improvement in disease management.

Detection of circulating tumor cells in patients with small cell lung cancer (SCLC) is a poor prognostic marker as well [94,95,96,97,98]. Different from other malignancies, higher rates of CTCs have been reported in SCLC. For example, a study of 60 patients with extensive stage SCLC identified CTCs in 90% of patients, with a range from 0 to 24,281 per 7.5 mL [99]. This same study reported that prognostic accuracy using CTCs detection was greatest in patients who had a reduction of CTCs count by 89% following chemotherapy. Other studies have supported the prognostic value of CTCs reduction following chemotherapy in SCLC [100,101]. Beyond the reduction in CTCs, monitoring tumor markers may also hold value in treatment navigation. One study evaluated vascular endothelial growth factor receptor (VEGFR) expression on CTCs of patients with SCLC undergoing treatment with pazopanib (VEGFR inhibitor), finding an initial reduction in CTCs expression with treatment initiation, but with disease progression, a significant increase in CTCs were observed with a significant increase in VEGFR expression [102]. This suggests a role for tailoring therapies to receptor expression of CTCs in SCLC.

#### 6.3.2. Precision Medicine

A recent prospective study evaluated CTC PD-L1 expression in patients with recurrent or metastatic NSCLC prior to and after initiation of ICI therapy and found that increased expression of CTC PD-L1 from prior treatment to after treatment was associated with better PFS and OS, which may potentially allow for determination of which patients may benefit from further ICI therapy [103]. In a study evaluating 30 patients with NSCLC without targetable mutations who had progression on first-line platinum-based chemotherapy and were now receiving sintilimab (PD-1 inhibitor) plus docetaxel, patients with high PD-L1 expression on CTCs had longer median PFS and longer median OS when compared to patients with low CTC PD-L1 levels [37]. These studies suggest a role for monitoring PD-L1 expression on CTCs in NSCLC to guide therapeutic decisions.

### 6.4. Colorectal Cancer

Within colorectal cancer, an active area of research surrounds the determination of which patients with stage II and stage III disease may benefit from adjuvant chemotherapy. As such, much of the research regarding the utility of CTCs has sought to evaluate a possible role in this population of patients.

#### 6.4.1. Prognostication

Among patients who are CTC-positive, quantification has been shown to help predict the extent of disease involvement. In a study of 121 patients with advanced colorectal cancer, of whom 71 were CTC-positive, CTC positivity was predictive of the depth of invasion, lymphatic involvement, distant metastatic disease, TNM staging, and serum CEA level, and was overall predictive of less favorable PFS and OS, with persistent presence during chemotherapy also associated with poorer PFS and OS [104]. Additional studies have correlated baseline CTC count *≥*3/7.5 mL with stage IV disease at diagnosis, at least three sites of metastasis, elevated CEA levels, and increased TNM staging [105,106]. Beyond disease characteristics, additional studies have supported the negative prognostication associated with CTC detection in both localized and advanced CRC prior to intervention [107,108,109,110].

As previously mentioned, an important question in colorectal cancer remains in regard to which patients benefit from adjuvant chemotherapy versus which patients may be able to be spared this intervention. A study published in 2019 suggested that post-operative CTC levels were more predictive of recurrence-free survival in patients with stage II–III CRC undergoing surgical resection [111]. However, conflicting results have also been published suggesting that CTC elevation following surgical resection was not predictive of patient outcomes [109,112]. This discrepancy may, in part, be due to the timing of sample analysis and represents an area where further study may be required.

However, CTCs do have prognostic value following chemotherapy in patients with stage III colon cancer, with a study demonstrating post-chemotherapy persistence correlated with worse DFS and OS [113]. An additional phase II trial enrolling patients with metastatic CRC found that persistently negative CTC status during chemotherapy predicted better OS [114].

#### 6.4.2. Treatment Guidance

An interesting phase II trial including 48 patients with advanced CRC who received a regimen of irinotecan, oxaliplatin, and tegafur-uracil—and made cross-trial comparisons to patients who received capecitabine, oxaliplatin, and bevacizumab +/− cetuximab—stratified patients by CTC count < 3 or *≥*3 and found that median OS was similar for both treatment groups if the baseline CTCs count was <3. However, patients receiving the regimen consisting of irinotecan, oxaliplatin, and tegafur-uracil had better survival when the CTC count was *≥*3 [38]. While no significant conclusions can be drawn based on the design of the study, the authors note that this is a hypothesis-generating study, suggesting that patients who have elevated CTC counts may benefit from more aggressive treatment regimens and allow for avoidance of higher toxicity chemotherapy in lower risk groups. 

This was followed by the phase III VISNU-1 trial, which was an open-label, phase III study that enrolled 349 patients with untreated, unresectable metastatic CRC with CTCs count *≥*3 and randomized them to receive FOLFOXIRI (irinotecan 165 mg/m^2^, oxaliplatin 85 mg/m^2^, leucovorin 400 mg/m^2^, and 5-fluorouracil 3200 mg/m^2^) plus bevacizumab or FOLFOX (oxaliplatin 85 mg/m^2^, leucovorin 400 mg/m^2^, 5-fluorouracil 400 mg/m^2^, then 2400 mg/m^2^) plus bevacizumab, and found that those who received FOLFOXIRI plus bevacizumab had longer PFS [115]. It is important to note that grade 3 toxicity was more frequently reported in the FOLFOXIRI arm. However, it is challenging to draw any conclusions from this study using a primary endpoint of PFS when comparing a triplet chemotherapy backbone to a doublet backbone, recognizing that a meaningful endpoint would be OS or time to second progression following receipt of a second doublet in those who received a doublet in the first line.

## 7. Surveillance

Currently, disease surveillance is accomplished via clinical examinations, imaging studies, and tumor markers. For patients who have elevated tumor markers at diagnosis, it is not uncommon to see an elevation of markers prior to the detection of the recurrent disease on imaging studies. However, current markers are lacking in sensitivity and specificity, particularly in the setting of minimal disease, demonstrating a need for better methods for early detection of minimal residual disease recurrence.

Circulating tumor cells may play a role in disease surveillance. In a study of prostate cancer patients undergoing active surveillance, CTCs positivity was noted to be a marker of upstaging and upgrading, and therefore, positive CTCs may be an indication for treatment in patients with a localized malignancy [116]. Conversely, in a study using CTCs for screening following surgical resection of early-stage breast cancer, the use of CTCs for disease monitoring was not associated with improved OS or DFS [117].

At this time, the use of CTC monitoring for recurrent disease is likely lacking due to the sensitivity of this assay. As laboratory techniques improve and sensitivity is improved, CTCs may be a viable assay for monitoring recurrent disease. 

## 8. CTCs vs. ctDNA

As previously mentioned, two primary biomarkers within liquid biopsy are CTCs and ctDNA, with each of these having unique strengths and limitations [4,118] (Table 3). While there is much discussion regarding which is the “better” assay, it is important to recognize that each is unique and provides different information regarding an individual malignancy, and perhaps, the optimal strategy may involve both—not one or the other.

CTC detection has demonstrated utility in cancer prognostication in malignancies, including breast, prostate, lung, and colorectal cancer, with persistently elevated levels despite therapy further predictive of poorer patient outcomes. With this in mind, there is likely a role in monitoring CTCs in response to therapy and adjusting treatment regimens in response, although trial data does not yet support this indication. Similar to ctDNA, CTCs allow for genomic analysis of malignancy, but beyond this, they also allow for phenotypic analysis of a heterogenous tumor population—for example, identifying PD-L1 expression, which may help predict response to immunotherapy. Additionally, CTCs have the potential to be cultured, allowing for patient-specific tumor models for therapy testing, which may have promising clinical applications in the future [119].

However, CTCs do have limitations. In a trial evaluating patients with colorectal cancer with liver metastases, ≥3CTCs/7.5 mL were detected in 19% of patients, whereas KRAS ctDNA detection used in this study was 91% sensitive [120]. At this time, the relatively low sensitivity of CTC assays remains a limitation of its use. In addition, CTCs are a heterogeneous population, and therefore, phenotypic expression may change as a result of treatment. While this may be a tool for monitoring treatment response and adjusting therapy, clinical trial data does not yet support this function.

Circulating tumor DNA, on the other hand, provides valuable, real-time data regarding tumor burden due to its short half-life, with studies demonstrating its superiority to CTCs in correlation with levels and tumor burden [121]. Beyond this, studies have demonstrated utility in monitoring for disease recurrence due to its high sensitivity and specificity, even in states of low disease burden [122]. Additionally, there is evidence supporting the utility of ctDNA in disease management. For example, in the case of non-small cell lung cancer, evidence suggests that EGFR T790M mutation status may be determined by ctDNA and used to guide therapy [123].

Like CTCs, ctDNA does have its limitations. First, ctDNA only allows for the genotypic characterization of malignancy, as opposed to CTCs, which allow for phenotypic analysis. In addition, the sensitivity of ctDNA may vary by organ involvement, with studies of recurrent metastatic CRC demonstrating higher sensitivity with hepatic metastasis over pulmonary metastasis [124].

## 9. Conclusions and Future Directions

CTCs are tumor cells that have detached from the primary tumor and entered the blood circulation, and have the potential to form metastatic disease. The study of CTCs has important implications not only for cancer diagnosis, prognosis, and treatments, but also for screening and surveillance. CTCs have been shown to be present in the blood of patients with almost all types of malignancy, and their detection and characterization can provide valuable information, particularly in regard to prognostication.

In the future, studies of CTCs will likely continue to play an important role in cancer research and clinical practice. However, several challenges still exist. CTC assays currently lack adequate sensitivity, especially in early-stage solid malignancies, due to low numbers of detectable CTCs [125]. One important area of research is the development of more sensitive and specific methods for detecting and analyzing CTCs. Recent advances in detection methods, including size-based detection, microfluidic, and nanotechnology, have enabled more precise and comprehensive characterization of CTCs.

Another area of future research is the functional analysis of CTCs, including their role in tumor progression, metastasis, and resistance to therapy. At this time, CTC analysis may play a role in cancer prognostication and phenotypic evaluation of tumor cells, but data regarding how to tailor treatment based on the phenotypic and genotypic expression of CTCs—as well as how to tailor treatment based on CTC response to therapy—is currently lacking. The molecular and cellular mechanisms that control CTC survival, migration, and cluster formation in distant organs are not yet fully understood and warrant further investigation. Furthermore, the potential clinical applications of CTCs are actively being explored. For example, CTC-based liquid biopsy has the potential to complement or replace traditional tissue biopsy, as it is less invasive and may provide real-time monitoring for disease progression, treatment response, and detection of newly acquired mutations in the cancer cells. Moreover, the identification of specific biomarkers for CTCs can aid in the development of targeted therapies that are tailored to individual patients (precision medicine).

As assays improve and more trials evaluate the clinical utility of CTC detection in guiding therapies, we anticipate increased use in cancer management. Full utilization of CTCs in the clinical management of malignancies—from cancer screening, diagnosis, and treatment navigation to surveillance—may be feasible in the near future. As of February 2023, over 80 trials involving CTCs in some capacity are actively recruiting (Table 4).

## Figures and Tables

**Table 1:** CTC isolation techniques.

	CTC Isolation Technique	Method	Advantages	Disadvantages
**Label dependent isolation**	Antibody conjugation to magnetic nanoparticles	Antibody linked to magnetic nanoparticles to isolate CTCs expressing specific marker	One assay with FDA approval; can use different antibodies to isolate different populations of cells	Low sensitivity; down-regulation of EpCAM markers during metastatic transformation can limit sensitivity
	Microfluidics	Controlled flow in microchip to enhance CTC binding to antibody coated microchip walls	High sensitivity with high cell viability	Only able to process small sample volumes
**Label independent isolation**	Filtration	Size-based separation with purification to isolate CTCs from other blood cells	Isolation regardless of surface marker expression	Requires large volumes; poor purity; pore clogging
	Microfluidics (not dependent on antibodies)	Flow through microchip to separate CTCs based on geometric properties	High sensitivity with high cell viability	Only able to process small sample volumes
	Density gradient separation	Centrifugation to separate CTCs from blood cells based on density	Efficient process; cell viability after isolation	Loss of cells (varying density when cells clump); often requires further isolation due to contamination with other blood cells
	Imaging	Fiber optic array laser scanning to visually detect CTCs	Enumeration of CTCs	Lacking precision
	Dielectrophoresis	Application of non-uniform electric field to isolate cells	High recovery rate and viability	Low purity of the isolated sample
	Inertial focusing	Fluid inertia at high flow rates to isolate cell populations	Recovery of viable cells	Requirement of pre-processing of sample

Abbreviations: CTC: circulating tumor cell; EpCAM: epithelial cell adhesion molecule.

**Table 2:** Pertinent clinical trials evaluating the clinical application of CTCs.

Malignancy	Trial Name	NCT Number	Patient Population	Study Arm	Control Arm	Patient Number	Study Outcome
Breast	N/A [33]	01185509	HER2-MBC w/HER2 + CTCs	Trastuzumab + vinorelbine	N/A	20	ORR 5%, mPFS 2.7 months
Breast	SWOG S0500 [34]	00382018	MBC w/persistent CTCs after 21 days of therapy	Continue initial therapy	N/A	123	mOS 10.7 vs. 12.5 months, *p* = 0.98
				Change chemotherapy			
Breast	STIC CTCs [35]	01710605	HR+, ERBB2-MBC	First line therapy by CTCs count (chemo if ≥5 CTCs/7.5 mL, endocrine if <5)	Clinician driven first line therapy	755	Noninferior OS (15.5 vs. 13.9 months, HR 0.94, 90% CI 0.81–1.09)
Prostate	PROPHECY [36]	N/A	mCRPC starting abiraterone or enzalutamide	CTCs AR-V7 status	N/A	118	Pretreatment detection of AR-V7 associated with poorer OS by two different assays (HR 3.3, 95% CI 1.7–6.3 and HR 3.0, 95% CI 1.4–6.3, respectively)
NSCL	N/A [37]	03798743	NSCL w/o targetable mutation w/progression after platinum-based chemo	Sintilimab plus docetaxol	N/A	30	Patients with high CTCs PD-L1 expression had better mPFS (6.0 vs. 3.5 months, *p* = 0.011) and mOS (15.8 vs. 9.0 months, *p* = 0.038)
CRC	N/A [38]	N/A	Advanced KRAS WT CRC	Irinotecan, oxaliplatin, and tegafur-uracil with leucovorin and cetuximab	N/A	48	mOS for CTCs ≥ 3/7.5 mL vs. <3 was 18.7 vs. 22.3 months (*p* = 0.038)

Abbreviations: MBC: metastatic breast cancer, CTCs: circulating tumor cell, ORR: objective response rate, mPFS: median progression-free survival, mOS: median overall survival, HR+: hormone receptor positive, HR: hazard ratio, 95% CI: 95% confidence interval, mCRPC: metastatic castration-resistant prostate cancer, AR-V7: androgen receptor splice variant 7, NSCL: non-small cell lung cancer, PD-L1: programmed death-ligand 1, CRC; colorectal cancer.

**Table 3:** The comparison (advantages and disadvantages) between CTC and ctDNA.

	Pros	Cons
**Circulating tumor cells**	Non-invasive Phenotypic and genotypic characterization of tumor cells Role in prognostication Creation of patient-derived xenograft	Current assays lacking in sensitivity Currently lacking trial data to guide treatment
**Circulating tumor DNA**	Non-invasive Genotypic characterization of malignancy Short half-life allows for real-time monitoring of tumor burden Evidence of role in guiding treatment	Inability to analyze tumor cell phenotype

**Table 4:** Selected ongoing clinical trials in CTCs and solid tumors.

Malignancy	Trial Name	NCT Number	Patients	Study Arm	Control Arm	Primary Outcome
CRC	POACC-1	03700411	Undergoing open radical surgery for CRC	Morphine	N/A	Change in CTCs count following surgery depending on the type of perioperative analgesia
				Piritramid		
				Epidural		
Breast	HER2Cell	04993014	Early HER2+ breast cancer w/complete response to neoadjuvant trastuzumab and pertuzumab	Adjuvant trastuzumab	N/A	DFS between adjuvant arms based on whether the patient had HER2 + CTCs at baseline analysis
				Adjuvant trastuzumab plus pertuzumab	N/A	
Liver	N/A	04800497	Resected HCC	N/A	N/A	Association between CTCs obtained 0, 30, 90, 180, and 365 days following surgical resection and DFS
Breast	N/A	03928210	Advanced or metastatic breast cancer	Digoxin	N/A	Change in CTCs cluster size after ingestion of oral digoxin
Breast	N/A	04065321	Luminal A breast cancer w/o lymph node involvement	CTCs monitoring	PET-CT examination	DFS
Prostate	C-ProMeta-1	05533515	Localized prostate cancer scheduled for robot-assisted prostatectomy	CTCs level prior to surgery and at 3 months post-surgery	N/A	Post-radical prostatectomy treatment failure during 4.5 years of follow-up
Thoracic malignancy	N/A	04048512	Neoplastic thoracic disease undergoing resection with intraoperative ECMO or CPB	CTCs quantification	N/A	Quantification of CTCs before and after surgery
			Neoplastic thoracic disease undergoing resection without ECMO or CPB			
Pancreas	EUS-CTCs	04677244	Suspected pancreas cancer	CTCs detection in portal venous blood before and after endoscopic biopsy	N/A	Frequency of increase of CTCs > 4cells/mL in portal system after endoscopic biopsy
Bladder	N/A	04811846	Recurrent transitional cell cancer of the bladder	Transurethral resection of bladder tumor	N/A	Change of CTC count in blood before, during, and after the procedure, and CTC number and characterization in purging fluid
				Plasma kinetic vaporization of bladder tumor		
Stomach	N/A	05208372	Gastric cancer	Radical laparotomy	N/A	Quantity and classification of CTCs in ascites and blood and expression of ctDNA in ascites and blood
				Laparoscope-assisted radical gastrectomy		

Abbreviations: CRC: colorectal cancer, CTCs: circulating tumor cell, DFS: disease-free survival, ECMO: extracorporeal membrane oxygenation, CPB: cardiopulmonary bypass.
